# The Cardiovascular Effects of Inflammatory Bowel Disease Therapy with Biologics and Small Molecules: A Comprehensive Review

**DOI:** 10.3390/jcm14186476

**Published:** 2025-09-14

**Authors:** Eleftheria M. Mastoridou, Fotios S. Fousekis, Xenofon M. Sakellariou, Konstantinos Mpakogiannis, Dimitrios N. Nikas, Lampros K. Michalis, Konstantinos H. Katsanos, Haralampos Milionis

**Affiliations:** 1 Division of Gastroenterology, Department of Internal Medicine, Faculty of Medicine, School of Health Sciences, University of Ioannina, 45500 Ioannina, Greece; e.mastoridou@uoi.gr (E.M.M.); fotisfous@gmail.com (F.S.F.); kostismpakogiannis@gmail.com (K.M.); 2 1st Division of Internal Medicine, Department of Internal Medicine, University Hospital of Ioannina, 45500 Ioannina, Greece; hmilioni@uoi.gr; 3 2nd Division of Cardiology, Department of Cardiology, University Hospital of Ioannina, 45500 Ioannina, Greece; xensakel@gmail.com (X.M.S.); lamprosmihalis@gmail.com (L.K.M.); 4 1st Division of Cardiology, Department of Cardiology, University Hospital of Ioannina, 45500 Ioannina, Greece; dimitrios.nikas@gmail.com

**Keywords:** inflammatory bowel disease, biologics, small molecules, cardiovascular safety, heart failure, arrhythmias, acute coronary syndromes

## Abstract

**Background/Objectives:** Ιnflammatory bowel disease (IBD), comprising Crohn’s disease (CD) and ulcerative colitis (UC), is increasingly associated with cardiovascular (CV) complications, such as heart failure (HF), arrhythmias, and acute coronary syndromes (ACSs). As the therapeutic landscape of IBD evolves, with the introduction of newer biologics and small molecules, their CV safety warrants critical evaluation. The objective of this review is to provide an update on the current evidence of CV risks associated with IBD treatments. **Methods:** A comprehensive literature search from inception to April 2025 was conducted using PubMed and Medline to identify randomized controlled trials, observational studies, systematic reviews, as well as pharmacovigilance data reporting CV safety outcomes of biologic and small-molecule drugs approved for IBD. Additionally, analysis of the European Summary of Product Characteristics for each agent was also performed. **Results:** Anti-TNF agents, particularly infliximab, have been associated with increased reporting of HF and arrhythmias, particularly in patients with pre-existing cardiac disease. Ustekinumab and vedolizumab show consistently favorable CV safety profiles across trials and real-world studies. IL-23p19 inhibitors demonstrate low CV event rates overall, although signals for atrial fibrillation have emerged with risankizumab. Janus kinase inhibitors and sphingosine-1-phosphate receptor modulators carry class-specific CV warnings, due to signals mainly on non-IBD populations, and require careful use in high-risk individuals. **Conclusions:** Although most IBD therapies are generally safe from a CV perspective, certain agents may pose risks in vulnerable patients. Individualized CV risk assessment and ongoing post-marketing surveillance are essential to guide therapeutic choices and ensure patient safety.

## 1. Introduction

Inflammatory bowel disease (IBD), comprising Crohn’s disease (CD) and ulcerative colitis (UC), is a chronic immune-mediated inflammatory disorder of the gastrointestinal tract (GI). Although IBD primarily affects GI, it is increasingly recognized as a systemic disease with extraintestinal manifestations (EIMs) that significantly impact patients’ quality of life [1]. In fact, growing evidence suggests that IBD patients are at increased risk for cardiovascular (CV) complications, including heart failure (HF), arrhythmias, and acute coronary syndromes (ACSs) [2]. The underlying mechanisms are multifactorial and include chronic inflammation, endothelial dysfunction, and potential adverse CV effects associated with certain IBD therapies [3].

Increasing evidence supports the link between IBD and adverse CV outcomes. Multiple studies have demonstrated that patients with IBD, especially during periods of active disease, have a significantly higher risk of CV events compared with the general population [2,4]. A recent meta-analysis highlighted subclinical abnormalities, such as subtle systolic and diastolic dysfunction, which may precede overt cardiac complications in IBD patients [2]. Furthermore, IBD patients exhibit a two-fold increased risk of HF associated with periods of disease activity [5].

The underlying pathogenetic mechanisms contributing to cardiac abnormalities in IBD are multifactorial and complex. There is substantial evidence that chronic inflammation along with immune dysregulation, genetic predisposition, and environmental factors can act both independently and synergistically to increase the risk of CV complications [6]. Chronic inflammation is believed to be a central driver, directly disrupting the delicate balance of endothelial function, by promoting vasoconstriction, leukocyte adhesion, and platelet activation, ultimately impairing vascular health [7]. Additionally, inflammation can disrupt lipid metabolism, leading to alterations in cholesterol levels and promoting the formation of atherogenic plaques [8].

Moreover, studies have demonstrated impaired coronary microvascular function in IBD patients, which is correlated with disease activity and duration [2]. This coronary microvascular dysfunction can contribute to myocardial ischemia (MI) and potentially increase the risk of adverse cardiac events. IBD can also affect the autonomic nervous system, which regulates heart rate (HR) and blood pressure (BP). Dysfunction of the autonomic nervous system can contribute to arrhythmias and other CV complications [9,10]. Given this increased baseline risk, clinicians should carefully assess IBD patients before initiating any therapy that could impact their CV health.

The management of IBD has evolved considerably in recent decades, mainly with the introduction of biologic agents and small molecules targeting specific inflammatory pathways. Biologic agents, such as anti-tumor necrosis factor (anti-TNF) agents and interleukin (IL) 12/23 inhibitors, target particular components of the immune system to reduce inflammation. More recently, selective IL-23 inhibitors have been approved for the treatment of IBD, offering additional targeted options for patients with moderate to severe disease [11,12,13]. Small-molecule drugs, including Janus kinase (JAK) inhibitors and sphingosine-1-phosphate (S1P) receptor modulators, act on intracellular signaling pathways that drive inflammatory responses [14]. Although these novel therapies have substantially advanced IBD management, their potential CV effects remain under active investigation, with some studies suggesting increased CV risk while others reporting no significant rise in CV events [2,3,7,15].

This review aims to critically evaluate emerging evidence on the potential cardiac effects of biologic agents and small molecules used in IBD. By analyzing findings mainly from clinical trials and real-world studies, we aim to clarify the potential cardiac risks and benefits associated with these therapies. Ultimately, evaluating the CV effects of IBD therapies, alongside the well-established pathophysiological mechanisms linking IBD to CV disease, will contribute to optimizing management strategies for clinical decision making.

## 2. Materials and Methods

### Literature Research

An in-depth review of the literature search was conducted using PubMed and Medline up to April 2025 to identify studies evaluating the CV effects of biologic agents and small molecules used in treating IBD. We use the following search string: (“ulcerative colitis” OR “Crohn’s disease”) AND (“infliximab” OR “adalimumab” OR “golimumab” OR “ustekinumab” OR “vedolizumab” OR “risankizumab” OR mirikizumab” OR “guselkumab” OR “filgotinib” OR “tofacitinib” OR “upadacitinib” OR “etrasimod” OR “ozanimod”) AND (“cardiovascular events” OR “heart failure” OR “arrhythmia OR “acute coronary syndrome”). Only studies published in English and involving adult populations were included in this review. The focus was on randomized controlled trials (RCTs), observational studies, systematic reviews, and pharmacovigilance data. Along with searching databases, we reviewed the Summary of Product Characteristics (SPC) for each approved therapy for IBD to identify any documented cardiovascular adverse events.

## 3. Cardiovascular Effects of Biologic Agents

### 3.1. Cardiovascular Effects of Anti-TNF Agents

TNF-α is a key cytokine in the pathogenesis of IBDs, as it promotes inflammation through downstream mediators such as interleukin (IL)-1b and IL-6 [16]. Among the TNF-α inhibitors, infliximab, adalimumab, and golimumab are commonly used in IBD treatment [17,18]. The development of biologic agents targeting TNF-α has revolutionized the treatment of moderate-to-severe CD and UC, particularly in patients unresponsive to conventional therapies [19]. Infliximab was the first anti-TNF agent approved for IBD, followed by adalimumab and golimumab, the latter being approved only for UC [20]. Despite their established effectiveness in promoting clinical and endoscopic responses, anti-TNF therapies are also associated with adverse events, including potential CV risks, particularly in patients with pre-existing heart conditions [21].

#### 3.1.1. Risk of Heart Failure Associated with Anti-TNF Therapy

Among the potential CV concerns, HF is particularly noteworthy and has led to class-wide warnings in the SPC for infliximab, adalimumab, and golimumab, especially in patients with pre-existing heart conditions [22,23,24]. More precisely, all the above agents are contraindicated in patients with moderate-to-severe symptomatic HF (New York Heart Association NYHA class III/IV). Additionally, they should be used with caution in patients with mild HF (NYHA I/II), as there have been reports of new-onset or exacerbated HF [22,23,24,25].

The primary source of cardiac safety concern, particularly for infliximab, stems from the ATTACH (Anti-TNF Therapy Against Congestive Heart Failure) trial, a placebo-controlled RCT involving non-IBD patients with moderate-to-severe HF [26]. This study found that infliximab, particularly at higher doses (10 mg/kg), worsened clinical outcomes and increased the risk of hospitalization. Although the standard IBD dose of 5 mg/kg did not demonstrate significant cardiac harm in the trial, the observed dose-dependent adverse effects prompted regulatory agencies to apply cautionary warnings to all dosing regimens, especially in patients with existing cardiac dysfunction.

Supporting the possibility of a dose- or time-dependent effect, subsequent studies have shown that short-term administration of infliximab can induce a transient rise in N-terminal pro-B-type natriuretic peptide (NT-proBNP) levels, a biomarker indicative of acute myocardial stress and an early marker of HF [27]. Importantly, long-term treatment with infliximab does not appear to result in sustained deterioration of cardiac function [27], suggesting that any deleterious effects may be limited to early treatment phases.

Data from IBD case reports suggest that anti-TNF therapy may be associated with both deterioration of pre-existing HF and new-onset HF, even in the absence of traditional risk factors. For instance, one case report documented that infliximab was potentially associated with reduced left ventricular ejection fraction (LVEF) after six months of treatment in a patient with CV risk factors [21]. However, Kwon et al. reported new-onset HF in a patient receiving infliximab, despite the absence of traditional risk factors [28].

Evidence regarding HF associated with adalimumab is mixed, but generally reassuring. While the SPC includes a warning for new or worsening HF, no significant signal has emerged from RCTs in IBD patients. In support of a neutral cardiac safety profile, a prospective observational study evaluated the effects of adalimumab on left ventricular function in patients with severe UC unresponsive to immunosuppressants. After three months of therapy, there were no significant changes in LVEF, left ventricular volumes, or global longitudinal strain (GLS), suggesting that adalimumab does not adversely affect myocardial performance in patients without pre-existing heart disease [29].

Pharmacovigilance data provide additional insights. A recent analysis of the U.S. Food and Drug Administration Adverse Event Reporting System (FAERS) evaluated 1041 cases of HF among 176,025 adverse events linked to biologic therapies in CD. Infliximab and adalimumab accounted for the majority of HF cases (48.1% and 41.8%, respectively), while newer biologics, such as ustekinumab and vedolizumab, were associated with a significantly lower frequency [30]. Although based on spontaneous reporting and subject to underreporting or reporting bias, the findings highlight that anti-TNFs, particularly infliximab, continue to be disproportionately associated with HF events compared with other biologics.

On the other hand, some data suggest potential cardioprotective effects of TNF-α inhibition. A clinical trial in IBD patients has shown that treatment with adalimumab and infliximab improved endothelial function, coronary microvascular flow, and myocardial deformation, likely by reducing systemic inflammation [31]. Similarly, another clinical trial in patients with RA reported increased LVEF and reduced serum levels of HF biomarkers, such as NT-proBNP, IL-6, and endothelin-1, following infliximab treatment, further supporting the possible cardioprotective effects of anti-TNF therapy [32].

#### 3.1.2. Risk of Arrhythmias Associated with Anti-TNF Therapy

Besides concerns about HF, reports have highlighted the potential for arrhythmogenic effects linked to anti-TNF therapy, especially infliximab, though solid evidence is limited. The SPCs for all three anti-TNFs mention that arrhythmias have been seen, usually shortly after infusion, mainly in patients with pre-existing CV disease [22].

In line with this context, a nationwide population-based study found that patients with IBD, especially those receiving biologic agents such as infliximab, have a higher risk of developing atrial fibrillation (AF) compared with the general population [33]. The mechanisms remain unclear but may involve cytokine-mediated myocardial irritation, infusion-related hypersensitivity, autonomic dysfunction, and TNFα effects on cardiac ion channels and myocardial remodeling [34]. Similarly, some case reports have described a range of conduction disturbances following anti-TNF therapy, which can occur early after infusion and are often self-limiting, but in some cases may require discontinuation of treatment. For instance, Sofos et al. reported a case of complete atrioventricular (AV) block in a CD patient following repeated infliximab infusions, which resolved upon drug discontinuation [35].

Interestingly, not all data points toward harm. In specific settings, anti-TNF agents may even exert stabilizing effects on cardiac electrophysiology. A study by Senel et al. demonstrated a statistically significant reduction in the corrected QT (QTc) interval after six months of infliximab treatment, suggesting a potential anti-arrhythmic effect, possibly related to the decrease in systemic inflammation [36].

Despite these mixed findings, the available evidence suggests that arrhythmias, while rare, may occur in susceptible individuals, especially shortly after infusion. As such, it may be prudent to monitor patients with known conduction disorders or prior arrhythmias more closely during and after administration of anti-TNF agents.

#### 3.1.3. Risk of Acute Coronary Syndromes Associated with Anti-TNF Therapy

ACSs, although not frequently reported, represent another potential CV concern associated with anti-TNF therapy. The SPCs for infliximab, adalimumab, and golimumab include mentions of MI as a possible adverse event, typically occurring shortly after administration [22,23,24]. These warnings mainly rely on post-marketing surveillance and isolated case reports, which have described rare but notable instances of ACSs following anti-TNF exposure.

Individual case reports have documented instances of ACSs, including MI, occurring shortly after initiation of anti-TNF agents. For instance, Panteris et al. reported a case of a patient with CD who developed a non-ST-elevation MI (NSTEMI) three days after the first infliximab infusion, without having any predisposing factors [37]. Similarly, Abedin et al. documented a case of ACS occurring during the administration of infliximab in a young patient with RA, notably in the absence of any pre-existing CV disease [38].

In addition to case-level evidence, real-world pharmacovigilance data support a possible link between TNF-α inhibitors and ischemic CV events. The analysis of the U.S. FAERS by Ma et al. [39] study found that adalimumab was significantly associated with increased reporting of ischemic events, including ΜΙ (reporting odds ratio [ROR] = 1.58, 95% confidence interval [CI]: 1.51–1.64). In contrast, infliximab, along with golimumab, did not show a significant disproportionality signal for ischemic events, suggesting that ischemic risk may differ across agents within the same therapeutic class [39].

Interestingly, while isolated ischemic events have been reported, large-scale studies currently do not support a clear link between anti-TNF therapy and a higher risk of MI. In fact, some observational studies in RA patients indicate that long-term TNF inhibition may lower systemic inflammation and improve overall vascular health. For example, a large cohort study by Dixon et al. demonstrated a lower risk of MI in RA patients who respond to anti-TNF-alpha therapy compared with non-responders [40]. However, due to limited IBD-specific data and the possibility of early post-infusion ischemic events, especially in individuals with established cardiovascular risk, continued vigilance is necessary. Pre-treatment CV risk assessment and careful monitoring during infusions can help reduce the likelihood of adverse events.

### 3.2. Cardiovascular Effects of IL-12/23 Inhibitors

The interleukin (IL)-12/23 pathway is crucial in the development of IBD by promoting the differentiation and maintenance of pro-inflammatory T cells [41]. Ustekinumab, a fully human monoclonal antibody that targets the p40 subunit shared by both IL-12 and IL-23, is approved for moderate-to-severe CD and UC in patients who have not responded to or cannot tolerate conventional therapy or anti-TNF agents [42,43]. Clinical trials have demonstrated that ustekinumab can induce clinical and endoscopic remission, promote mucosal healing, and maintain long-term response with a favorable safety profile [42,43].

While these studies have confirmed its therapeutic benefits, data on the CV safety of ustekinumab are still limited. Real-world evidence from a prospective two-year Swedish cohort reported no CV adverse events among CD patients treated with ustekinumab, further supporting the absence of CV signals in trial settings [44]. Although CV outcomes were not a primary endpoint, the absence of reported events over the extended follow-up period reinforces its favorable safety profile in routine clinical practice. Despite this reassuring safety data, the potential CV effects of ustekinumab should be carefully evaluated, especially in patients with pre-existing CV comorbidities.

#### 3.2.1. Risk of Heart Failure Associated with IL-12/23 Inhibitors

HF has not been identified as a common or expected adverse event in patients receiving ustekinumab. According to the European SPC, HF is not listed as a common, serious, or dose-related adverse effect, and there are no contraindications for patients with underlying cardiac dysfunction [45]. Clinical trials in IBD, including the pivotal UNITI-1, UNITI-2, IM-UNITI, and UNIFI studies, reported no cases of HF and found no evidence of increased CV risk compared to a placebo [42,43].

Real-world evidence supports these findings. A large multicenter retrospective cohort study conducted by the SUCCESS Consortium, which included over 1100 CD patients in routine clinical practice, reported no new safety signals compared to clinical trials and no cases of HF as emergent adverse events [46]. Supporting this, the SUSTAIN study, a large Spanish real-world cohort involving 463 CD patients, also reported no cases of HF during long-term ustekinumab therapy [47].

Furthermore, a multicenter observational retrospective study by Teresa et al. evaluating ustekinumab in bio-naïve CD patients reported that four individuals with a history of congestive HF initiated ustekinumab without experiencing any cardiac adverse events during follow up, further supporting the cardiac tolerability of the drug in real-world settings [48].

#### 3.2.2. Risk of Arrhythmias Associated with IL-12/23 Inhibitors

To date, no association has been established between ustekinumab and arrhythmias. Neither the SPC nor clinical trials have reported cases of cardiac conduction disorders associated with ustekinumab use, supporting a consistent CV safety profile over time [42,43].

Additionally, no signal for arrhythmias has emerged from real-world databases, including recent pharmacovigilance analyses from the U.S. FAERS [39]. This absence of signals in IBD is further supported by real-world comparative data from a large U.S. claims cohort of biologic-naïve psoriasis patients, in which the rate of new-onset AF was essentially identical between those receiving ustekinumab and those on TNF-α inhibitors [49]. Although derived from a non-IBD population, this parallel finding reinforces that ustekinumab does not confer an elevated arrhythmia risk relative to other biologic therapies.

#### 3.2.3. Risk of Acute Coronary Syndromes Associated with IL-12/23 Inhibitors

Although CV events have been evaluated in both clinical trials and post-marketing studies of ustekinumab, ACSs, including MI and unstable angina, are not explicitly listed as known or expected adverse reactions in the SPC [45]. In the UNIFI trial for UC, only a single MI occurred over more than 900 patient-years of exposure [43]. Similarly, a pooled safety analysis of six phase 2/3 trials in CD and UC, involving over 4800 patient-years of exposure, evaluated the overall safety profile of ustekinumab, including the incidence of MACE, such as non-fatal MI. The rates of MACE were low and comparable to those in the general population, further supporting the CV safety of ustekinumab [50].

Post-marketing surveillance showed no disproportionality signal for MI or coronary thrombosis with ustekinumab, in contrast to elevated signals observed with specific TNF-α inhibitors, such as adalimumab [39]. In a real-world cohort study presented at the 2025 Crohn’s & Colitis Congress, ustekinumab was associated with a significantly lower risk of all-cause mortality, coronary revascularization, and non-ST-elevation myocardial infarction (NSTEMI) compared with vedolizumab in older adults with IBD [51]. However, no significant difference in acute MI incidence was observed between the two treatments [51].

While data from IBD populations remain reassuring, findings from other patient groups emphasize the need for vigilance. A French case-time-control analysis of psoriasis patients reported increased odds of ACSs within the first six months of ustekinumab initiation among individuals with a high baseline CV risk (OR 4.17; 95% CI 1.19–14.59) [52]. Although derived from a dermatologic cohort, this finding underscores the importance of individualized cardiac risk assessment and close monitoring when initiating ustekinumab in elderly patients or those with known coronary artery disease.

### 3.3. Cardiovascular Effects of IL-23p19 Inhibitors

Unlike ustekinumab, which targets the shared p40 subunit of IL-12 and IL-23, the recently approved IL-23p19 inhibitors, guselkumab, mirikizumab, and risankizumab, selectively block IL-23 signaling. This selective inhibition suppresses Th17-driven inflammation while preserving IL-12–mediated immunity [53]. These agents have demonstrated efficacy in inducing and maintaining clinical and endoscopic remission in moderate-to-severe UC and CD, supported by robust data from pivotal clinical trials [11,54,55]. With these promising results, attention has increasingly focused on their long-term safety profiles, including their potential CV effects, which, notably, are not listed as identified risks in the respective SPCs [56,57,58].

#### 3.3.1. Risk of Heart Failure Associated with IL-23p19 Inhibitors

Across RCTs and integrated safety analyses of mirikizumab, risankizumab, and guselkumab in IBD, there have been no significant safety signals or consistent reports of HF, and no recognized risk has been identified in the respective SPCs. In the FDA’s Multidiscipline Review for Risankizumab, CV events, including HF, occurred at low rates, suggesting no meaningful increase in CV risk with risankizumab use [59]. Complementary pharmacovigilance data from the FAERS have not reported a statistically significant association between HF and the use of risankizumab or guselkumab [60]. On the contrary, a pooled safety analysis in psoriasis populations did report an isolated case of death due to congestive HF in a patient with multiple CV risk factors, assessed by the investigator as possibly related to risankizumab treatment [61]. However, these collective findings support the conclusion that HF remains an uncommon event in patients treated with IL-23p19 inhibitors, although ongoing real-world monitoring remains prudent to detect any emerging CV signals.

#### 3.3.2. Risk of Arrhythmias Associated with IL-23p19 Inhibitors

No cases of arrhythmias or conduction abnormalities have been systematically reported in RCTs evaluating mirikizumab, risankizumab, and guselkumab in IBD, nor are they mentioned in their SPCs. However, recent pharmacovigilance data from the FAERS database indicate a significant safety signal for AF with risankizumab use, suggesting a potential association with this arrhythmia in real-world practice [60]. Conversely, guselkumab has not been associated with a statistically significant increase in AF reporting despite a small number of identified cases [60]. These findings underscore the need for continued vigilance regarding mainly the CV safety of risankizumab, particularly in patients with pre-existing risk factors for AF.

#### 3.3.3. Risk of Acute Coronary Syndromes Associated with IL-23p19 Inhibitors

Although CV events have been evaluated in both clinical trials and post-marketing studies of ustekinumab, ACSs, including MI and unstable angina, are not specifically listed in the SPC as known or expected adverse reactions [45]. In the UNIFI trial for UC, only a single MI occurred over more than 900 patient-years of exposure to ustekinumab [43]. Similarly, a pooled safety analysis of six phase 2/3 trials in CD and UC, involving over 4800 patient-years of exposure, assessed the overall safety profile of ustekinumab, including the incidence of MACE such as non-fatal MI. The rates of MACE were low and similar to those in the general population, further supporting the CV safety of ustekinumab [50].

Post-marketing surveillance showed no disproportionality signal for MI or coronary thrombosis with ustekinumab, unlike the elevated signals observed with certain TNF-α inhibitors, such as adalimumab [39]. In a real-world cohort study presented at the 2025 Crohn’s & Colitis Congress, ustekinumab was associated with a significantly lower risk of all-cause mortality, coronary revascularization, and non-ST-elevation myocardial infarction (NSTEMI) compared with vedolizumab in older adults (>50 years) with IBD [51]. However, there was no significant difference regarding MACE, including the incidence of acute MI, between the two treatments [51].

While data from IBD populations remain reassuring, findings from other patient groups highlight the need for vigilance. A French case-time-control analysis of psoriasis patients reported increased odds of ACSs within the first six months of ustekinumab initiation among individuals with a high baseline CV risk (OR 4.17; 95% CI 1.19–14.59) [52]. Although derived from a dermatologic cohort, this finding underscores the importance of individualized cardiac risk assessment and close monitoring when initiating ustekinumab in elderly patients or those with known coronary artery disease.

### 3.4. Cardiovascular Effects of the α4β7-Integrin Inhibitor

Integrin α4β7 mediates leukocyte trafficking to the gut by binding to mucosal addressin cell adhesion molecule-1 (MAdCAM-1), a pathway that is selectively upregulated in IBD. Vedolizumab is a humanized monoclonal antibody against α4β7 integrin that blocks lymphocyte homing to the intestinal mucosa without suppressing systemic immunity [62]. Vedolizumab has received regulatory approval for treating moderate-to-severe UC and CD in patients who do not respond adequately to standard therapies, based on results from the GEMINI 1 and GEMINI 2 trials [63,64]. These clinical trials have shown that vedolizumab induces clinical and endoscopic remission, promotes mucosal healing, and sustains long-term response with a favorable safety profile [63,64].

Post-marketing experience and real-world data continue to support the overall safety of vedolizumab. A 2025 pharmacovigilance analysis of over 46,000 adverse event reports from the FAERS database identified ‘cardiac disorders’ as a reported system organ class, but with a lower-than-expected signal strength (ROR 0.44), suggesting no elevated CV risk [65]. Likewise, the VICTORY Consortium, a large U.S.-based real-world registry, has not identified cardiac events as prominent safety signals during vedolizumab treatment [66].

#### 3.4.1. Risk of Heart Failure Associated with Vedolizumab

HF has not been identified as a safety concern with vedolizumab. The SPC does not list HF among known adverse reactions, nor is there a contraindication for patients with pre-existing cardiac dysfunction [67]. No HF events were reported in the pivotal GEMINI trials [63,64], nor in the long-term GEMINI LTS study, which included over 2200 patients and 8000 patient-years of exposure [68].

Real-world data from the ENEIDA registry, which included 521 patients with IBD treated with vedolizumab, reported three HF cases, corresponding to an incidence rate of 0.5 per 100 patient-years [69]. Although the registry was not designed to evaluate CV outcomes in detail, the low frequency of HF supports the view that it remains a rare event in vedolizumab-treated patients. Notably, no cardiovascular safety signal has emerged to date in real-world analyses or long-term follow-up studies.

#### 3.4.2. Risk of Arrhythmias Associated with Vedolizumab

To date, vedolizumab has not been linked to a higher risk of arrhythmias in either clinical trials or observational studies. In the key GEMINI 1 and 2 trials and their long-term extension (GEMINI LTS), no conduction issues or electrocardiographic abnormalities were observed among vedolizumab-treated patients [63,64,68].

Similarly, no arrhythmic events have been reported in large real-world registries. A single case of complex arrhythmia was documented in the ENEIDA registry in a patient with pre-existing AF and ischemic heart disease; however, the event was not attributed to vedolizumab itself [69]. Additionally, a pharmacovigilance analysis using the U.S. FAERS showed a slightly higher-than-expected rate of heart rate changes in IBD patients treated with vedolizumab compared with anti-TNFs [70]. While these findings do not indicate a direct arrhythmic risk, they highlight the importance of ongoing monitoring for non-specific cardiac rate abnormalities, especially in patients with pre-existing CV conditions. To our knowledge, no published case reports have directly linked vedolizumab to new-onset arrhythmias, further supporting its favorable cardiac rhythm safety profile.

#### 3.4.3. Risk of Acute Coronary Syndromes Associated with Vedolizumab

ACSs have not emerged as a consistent safety concern in patients treated with vedolizumab. The European SPC for vedolizumab does not list ACSs under cardiac disorders, confirming that no ischemic cardiac events occurred with sufficient frequency during clinical development or early post-marketing surveillance to warrant a standalone listing [67]. A fatal case of ACS occurred in a 66-year-old man shortly after a single dose of vedolizumab in GEMINI 1; the investigators did not establish a causal link, but the event was documented as a serious adverse event [63]. In GEMINI 2, no ACS events were reported in either the induction or maintenance phases [64].

Post-marketing safety data, including long-term extension trials and real-world pharmacovigilance analyses, have not identified a clear signal for ACSs in patients treated with vedolizumab. However, a real-world propensity-matched cohort study found that older IBD patients treated with vedolizumab had a higher risk of all-cause mortality, coronary revascularization, and NSTEMI compared with those receiving ustekinumab [51]. These findings emphasize the importance of individualized CV risk assessment when initiating biologic therapy in older adults, particularly those with multiple CV risk factors.

Table 1 provides a comparative overview of CV safety considerations for all approved biologic agents used in IBD, based on evidence from SPCs, clinical trials, and pharmacovigilance databases. It emphasizes agent-specific data related to HF, ACSs, and arrhythmias.

## 4. Cardiovascular Effects of Small Molecules

### 4.1. Cardiovascular Effects of JAK Inhibitors

Small-molecule JAK inhibitors such as tofacitinib (pan-JAK), upadacitinib, and filgotinib (both selective for JAK1) effectively inhibit pro-inflammatory cytokine signaling in moderate-to-severe UC. All three are approved for UC, while upadacitinib also has regulatory approval for CD [73].

While revolutionizing treatment efficacy, their CV safety has attracted attention due to signals in other immune-mediated diseases, especially RA, prompting regulators to issue warnings for serious heart-related events [74]. As outlined in the SPCs of JAK inhibitors, in patients aged 65 or older, current or past long-term smokers, and those with a history of atherosclerotic CV disease or other CV risk factors, JAK inhibitors should only be used if no suitable treatment options exist [75,76,77]. Therefore, in the context of IBD, clinicians need to balance inflammation control, which is independently linked to CV risk, with monitoring for potential off-target CV effects.

#### 4.1.1. Risk of Heart Failure Associated with JAK Inhibitors

HF has not been prospectively assessed as a predefined outcome in RCTs or observational studies of JAK inhibitors in IBD. The SPC for tofacitinib lists peripheral oedema as a common adverse event [75], a consideration potentially relevant to HF exacerbation in patients with pre-existing CV comorbidities due to fluid retention.

Although HF-specific data are lacking, composite CV outcomes may provide indirect insights. A 2024 real-world, propensity-matched cohort study of IBD patients aged 50 or older with CV comorbidities reported no significant difference in the incidence of acute HF between JAK inhibitor and TNF inhibitor users (0.76 [95% CI: 0.33–1.75] [78]). However, given the known HF signal with anti-TNF agents, similar vigilance may be prudent when prescribing JAK inhibitors, especially in patients with existing cardiac dysfunction.

#### 4.1.2. Risk of Arrhythmias Associated with JAK Inhibitors

The current evidence does not indicate an association between JAK inhibitor therapy and arrhythmias or conduction issues in IBD. In the OCTAVE program, arrhythmia-related events such as palpitations, extrasystoles, and sinus tachycardia were rarely reported in patients treated with tofacitinib, with no serious or high-grade conduction problems [79]. However, SPCs or regulatory agencies do not list rhythm disturbances as known side effects. Post-marketing data have also not indicated a signal of arrhythmic risk in this group.

#### 4.1.3. Risk of Acute Coronary Syndromes Associated with JAK Inhibitors

ACSs, including MI, have not consistently emerged as a safety signal in clinical trials of JAK inhibitors for IBD. In the OCTAVE clinical program evaluating tofacitinib in UC, adjudicated CV events were rare but included isolated cases of ACS, coronary artery stenosis, and MI [79]. Notably, the single MI case occurred in a patient receiving tofacitinib at maintenance doses. These findings emphasize the importance of individualized CV risk assessments, regardless of dose, especially in patients with pre-existing risk factors.

While the SPCs for all approved JAK inhibitors includes class-wide warnings for MACE, these primarily stem from the ORAL Surveillance trial, a key post-marketing study in RA patients aged ≥50 years with pre-existing CV risk [80]. In this high-risk group, tofacitinib showed a numerically higher incidence of MACE, especially MI, compared with anti-TNFs. Although the hazard ratio for MACE (HR 1.33; 95% CI, 0.91–1.94) did not reach statistical significance, the results led to a class-wide update in product labeling and the issuance of black-box warnings for all JAK inhibitors. Conversely, a Bayesian network meta-analysis of 26 randomized IBD trials (10,537 patients) found no statistically significant increase in MACE, though trends at higher doses (e.g., tofacitinib 5 mg BID, upadacitinib 30 mg QD) suggested a potential signal needing further research [81].

In real-world analyses, findings have been reassuring. A propensity-matched cohort of IBD patients ≥ 50 years with CV comorbidities found no significant difference in MACE, including MI, between those receiving JAK inhibitors and TNF inhibitors over 12 months (adjusted OR 0.92; 95% CI 0.41–2.03) [78].

### 4.2. Cardiovascular Effects of S1P Receptor Modulators

S1P receptor modulators, such as ozanimod and etrasimod, are oral agents used for moderate-to-severe UC that reduce intestinal inflammation by selectively blocking S1P_1_-mediated lymphocyte egress from lymphoid tissues [82]. These drugs were designed as more selective S1P receptor modulators to improve tolerability and minimize off-target effects. However, since S1P receptors are also present in CV tissues, their use raises potential concerns about bradyarrhythmia, conduction disturbances, and vascular events. Therefore, due to their possible cardiac effects, it is essential to evaluate the cardiovascular safety of ozanimod and etrasimod through evidence from clinical trials, regulatory documentation, and real-world data.

#### 4.2.1. Risk of Heart Failure Associated with S1P Receptor Modulators

Although S1P receptors are involved in vascular tone and cardiac function, neither ozanimod nor etrasimod has demonstrated an increased risk of HF in clinical trials. In a pooled post hoc analysis of the TRUE NORTH phase 3 trial and its open-label extension (OLE), which together included over 2200 patient-years of ozanimod exposure, no cases of new-onset or worsening HF were observed [83]. Similarly, the pooled safety data from ELEVATE UC 12 and 52, the pivotal trials of etrasimod, did not identify any HF-related adverse events [84].

Nonetheless, product labels for both drugs advise against use in patients with recent decompensated or NYHA class III–IV HF, reflecting a class-wide precaution [85,86]. However, to date, no HF signal has appeared from real-world experience. A 2024 observational study of ozanimod-treated patients over 12 months showed no HF events or CV-related discontinuations [87]. Data on etrasimod in real-world settings is limited but aligns with the absence of HF risk observed in clinical trials.

#### 4.2.2. Risk of Arrhythmias Associated with S1P Receptor Modulators

Given that S1P receptors are expressed in the sinoatrial and AV nodes, where they modulate heart rate and conduction, S1P modulators carry specific warnings regarding cardiac conduction disturbances. The SPCs for both ozanimod and etrasimod contraindicates their use in patients with severe, untreated conduction abnormalities (e.g., Mobitz type II second-degree or third-degree AV block) in the absence of a functioning pacemaker [85,86]. Additionally, a baseline electrocardiogram (ECG) prior to initiation is highly recommended, particularly in patients with known bradycardia or pre-existing conduction disorders.

These warnings are supported by findings from pivotal clinical trials. In the TRUE NORTH study, ozanimod was administered using a 7-day dose-escalation regimen to mitigate first-dose bradycardia, a known class effect of S1P modulators [88]. A dedicated CV safety analysis of TRUE NORTH and its open-label extension reported a mean HR decrease of 0.2 bpm on day 1, with two cases of asymptomatic bradycardia and no Mobitz type II or higher-degree AV block over more than 2200 patient-years of exposure [83]. In the ELEVATE UC 52 and UC 12 trials of etrasimod, bradycardia and AV block were infrequent, non-serious, and primarily occurred during treatment initiation [84]. No Mobitz type II or higher AV blocks or serious bradycardia events were reported, and all conduction abnormalities resolved without intervention.

More recent analyses reinforce the favorable arrhythmia profile of these agents. A 2023 prospective real-world study of ozanimod in UC reported no episodes of symptomatic bradycardia and no new safety signals over 12 months of follow up [87]. Comparative CV safety data remain limited for etrasimod, but a pooled analysis of ELEVATE and phase 2 trials reported low incidences of bradycardia and AV block primarily occurring on day 1 of treatment. All events were non-serious, transient, and resolved without intervention [14].

Accordingly, a recent Delphi consensus classified S1P receptor modulators as having a low CV risk, citing the absence of MACE or arrhythmic signals in trials and supporting routine baseline ECG screening, particularly in patients with pre-existing cardiac conditions [4].

#### 4.2.3. Risk of Acute Coronary Syndromes Associated with S1P Receptor Modulators

Use of ozanimod and etrasimod is also contraindicated in patients with a recent history, within the past six months, of MI or unstable angina reflecting a class-wide precaution outlined in both drugs’ SPCs [85,86]. Notably, both the TRUE NORTH and ELEVATE UC programs excluded patients with recent MI, unstable angina, or other significant CV conditions [84,88]. This restricted enrollment likely contributed to the absence of events in the trial data and may partly explain why the SPCs for both ozanimod and etrasimod contraindicate their use in patients with recent acute coronary syndromes.

In the integrated safety analysis of the etrasimod UC clinical program, one case of coronary artery disease (CAD) was reported; however, it was considered non-serious and did not result in treatment discontinuation [14]. Nevertheless, real-world CV safety data are currently lacking, underscoring the need for further studies in IBD populations.

Table 2 provides a comparative overview of CV safety considerations across all approved small molecules used in IBD, based on evidence from SPCs, clinical trials, and pharmacovigilance databases. It highlights agent-specific data related to HF, ACSs, and arrhythmias.

## 5. Conclusions and Perspectives

CV safety is increasingly recognized as a crucial factor in the long-term management of IBD. Chronic systemic inflammation is a well-known driver of atherosclerosis, HF, and arrhythmias, and IBD seems to share this underlying pathophysiologic process. Recent data indicate that patients with active IBD are at a significantly higher risk for adverse CV events, including MACE, autonomic dysfunction, and microvascular impairment. With the advent of targeted biologics and small-molecule therapies, clinicians must carefully assess the effectiveness of new treatments while considering potential off-target risks, aiming to control inflammation effectively while minimizing the risk of CV adverse effects.

Among currently approved therapies, anti-TNF agents remain central to IBD management but present a nuanced CV profile. Infliximab, in particular, is associated with new-onset or exacerbation of HF, especially in patients with pre-existing cardiac disease. Evidence from non-IBD populations, such as the ATTACH trial, and real-world pharmacovigilance data supports this concern. Although some studies report improvements in endothelial function and myocardial performance with anti-TNF therapy, conflicting data and post-marketing reports of arrhythmias and ACSs highlight the need for pre-treatment cardiac risk assessment. Mechanistically, TNF-α exerts complex, dose-dependent effects on the myocardium: low-level signaling may be cardioprotective, whereas sustained elevations promote apoptosis, oxidative stress, and adverse remodeling [89]. Inhibition of TNF-α can therefore disrupt homeostatic signaling, particularly via differential engagement of TNF receptor-1 (TNFR1) and TNFR2, potentially predisposing to HF in vulnerable individuals [90].

Conversely, ustekinumab and vedolizumab have consistently shown favorable CV safety profiles in both clinical trials and real-world settings. These agents are therefore regarded as appealing options for patients with high CV risk, providing targeted intestinal effectiveness without significant systemic CV effects. Their mechanisms partly explain this reassuring profile: ustekinumab selectively targets the p40 subunit of the IL-12/23 axis without interfering with cytokines directly involved in vascular remodeling or endothelial health [41]. Additionally, vedolizumab’s gut-specific mechanism reduces systemic immune suppression and off-target cardiovascular effects [91]. Similarly, early data on IL-23p19 inhibitors (guselkumab, mirikizumab, and risankizumab) indicate a reassuring CV profile. Notably, preclinical studies show that anti-IL-23p19 therapy lowers inflammatory vascular cytokines such as IL-17A, IL-6, and TNF-α in atherosclerosis-prone mice, suggesting possible anti-atherogenic effects [92]. However, long-term observational studies are still required to confirm their safety, especially in older adults or those with subclinical CV disease.

On the contrary, small-molecule therapies, such as JAK inhibitors and S1P receptor modulators, include class-specific CV concerns. Although JAK inhibitors carry black-box warnings related to MACE largely based on RA data, emerging evidence suggests that the absolute risk may be reduced, especially with proper patient selection and dosage management. Nonetheless, the JAK–STAT pathway plays a central role in vascular inflammation, atherogenesis, and thrombosis by modulating cytokine signaling and platelet activation; its inhibition may also alter lipid metabolism [93]. S1P receptor modulators are effective in UC but are linked to transient bradycardia and AV conduction abnormalities, particularly at the onset of treatment, requiring baseline cardiac evaluation and careful administration in patients with conduction disorders or recent ischemic events. Mechanistically, their functional antagonism causes S1P_1_ receptor internalization, reducing receptor signaling in sinoatrial and atrioventricular nodal tissue and triggering transient bradyarrhythmias [94].

**Figure 1:**
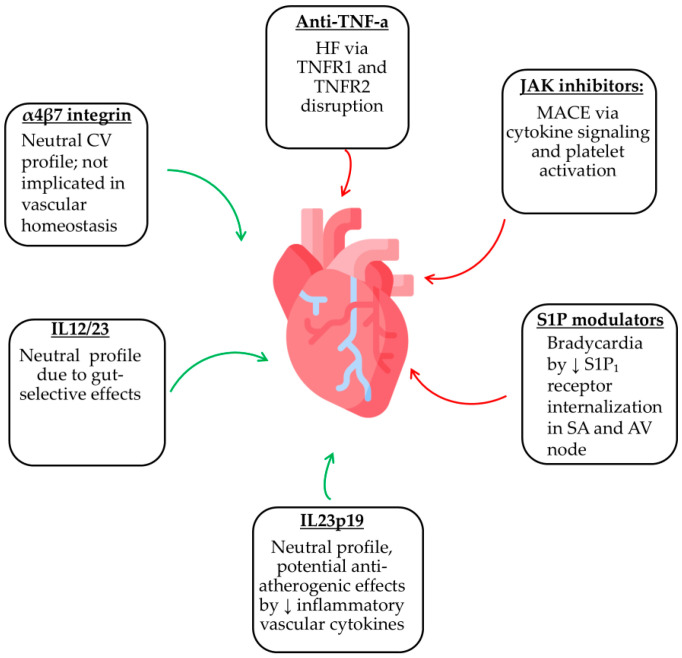
Cardiovascular effects of IBD therapies. Anti-TNFs may increase the risk of heart failure (HF), JAK inhibitors are associated with major adverse cardiovascular events (MACE), and S1P modulators can cause bradycardia and conduction abnormalities. In contrast, IL-12/23, IL-23p19, and α4β7 integrin inhibitors may have neutral or even beneficial cardiovascular profiles, with IL-23p19 agents possibly providing anti-atherogenic effects.

Aside from the well-documented therapies mentioned above, probiotics such as Escherichia coli Nissle 1917, although not part of advanced biologic or small-molecule treatments, are occasionally used as auxiliary treatments in IBD [95]. Current evidence does not suggest direct cardiovascular toxicity. Any potentially beneficial effects seem to be indirect, mainly through microbiome modulation and reduction of systemic inflammation, which may support cardiovascular health [96]. Figure 1 shows the CV safety profiles of currently approved IBD therapies, highlighting both potential risks and reassuring signals across different drug classes.

Improvement of CV outcomes in patients with IBD requires the incorporation of two synergistic approaches: successfully controlling chronic intestinal inflammation, which contributes to vascular dysfunction, and proactively identifying and managing CV modifiable risk factors. A ‘one-size-fits-all’ approach is no longer acceptable. Instead, a precision medicine framework, incorporating disease activity, comorbidities, age, CV history, and medication-specific risks, is essential in the management of IBD patients.

Notably, despite increasing awareness, several research gaps remain. The lack of validated CV risk stratification tools tailored for IBD populations and the limited long-term real-world safety data for new therapies, including IL-23p19 inhibitors, JAK inhibitors, and S1P modulators, are significant issues. Additionally, the variation in study designs, endpoints, and populations complicates direct comparisons between agents. Clinical trials often exclude high-risk individuals and are underpowered to detect rare cardiac events. Therefore, real-world data, such as pharmacovigilance reports and prospective registries, are essential for a comprehensive safety assessment. Importantly, patient advocacy and shared decision making should direct treatment choices, especially when weighing the minor but potentially serious CV risks against the benefits of disease management.

A multidisciplinary approach will be essential in selecting personalized therapy, guided by individual CV profiles. As the therapeutic landscape continues to grow, cardiogastroenterology will likely become a key interdisciplinary field in improving care for IBD patients.

## Figures and Tables

**Table 1:** Comparative CV safety of biologics.

Biologics	SPC Warnings	Clinical Trial Evidence	Real-World Data
Infliximab (Anti-TNFα)	Contraindicated in NYHA III/IV HF; caution in NYHA I/II; reports of myocardial ischemia and arrhythmia data	ATTACH (non-IBD): HF at high dose; Kotyla [32] et al. (2012): ↑LVEF, ↓NT-proBNP [26]; Senel et al. (2011) [36]: ↓QTc interval	Disproportionate HF reporting with infliximab in CD [30]; no disproportionality signal for HF, MI, or arrhythmias (i.e., AF) with infliximab [39]
Adalimumab (Anti-TNFα)	Same as infliximab; includes warning for new/worsening HF, reports of myocardial ischemia and arrhythmia	No change in LVEF or GLS [29]; RA patients: ↓MI risk in responders [40]; no arrhythmia trial data	Adalimumab accounted for 41.8% of HF reports; lower HF event ratio vs infliximab, but the highest HF-related hospitalizations [30]; no signal for HF or arrhythmias (i.e., AF); positive disproportionality signal for ACSs [39]
Golimumab (Anti-TNFα)	Same HF warnings; reports of myocardial ischemia and arrhythmia	No HF-specific RCTs or ACS/arrhythmia-focused studies	No disproportionality signal for HF, ACSs, or arrhythmias (i.e., AF) [39]; post-marketing safety data on cardiac events are very limited
Ustekinumab (IL-12/23 inhibitor)	No CV contraindications	Incidence of MACE (including non-fatal MI) was similar between ustekinumab and placebo [50]	SUCCESS Consortium; SUSTAIN study: No HF, arrhythmia, or ACS events reported No disproportional signal for HF, arrhythmia, or ACSs [39]; lower risk of all-cause mortality, NSTEMI in older IBD adults compared with vedolizumab [51]
Guselkumab, Mirikizumab, Risankizumab (IL-23p19 inhibitors)	No CV warnings or contraindications; no HF, ACSs, or arrhythmia listings in SPCs	No HF cases; 1 MI in FORTIFY (non-drug-related) [71]; no arrhythmias reported; LUCENT-3: few cerebrovascular events, not specified as ACSs [72]	AF and coronary signal with risankizumab in FAERS [60]; no disproportional signal for HF with guselkumab or risankizumab [60]
Vedolizumab (α4β7 integrin inhibitor)	No CV warnings or contraindications	GEMINI trials: no HF or arrhythmias reported; 1 ACS case without established causality [63,64],	Low reporting rates for cardiac events in FAERS [65]; ENEIDA registry: 3 cases of HF [69]; slightly higher-than-expected reports of changes in HR with vedolizumab than with anti-TNFs [70]; higher risk of all-cause mortality, NSTEMI compared with ustekinumab in older adults [51]

CV, cardiovascular; HF, heart failure; MI, myocardial infarction; ACSs, acute coronary syndromes; AF, atrial fibrillation; FAERS, Food and Drug Administration Adverse Event Reporting System; NSTEMI, non-ST-elevation myocardial infarction; HR, heart rate; IBD, inflammatory bowel disease; anti-TNF, anti-tumor necrosis factor; IL, interleukin; SPC, Summary of Product Characteristics.

**Table 2:** Comparative CV safety of small molecules.

Small Molecules	SPC Warnings	Clinical Trial Evidence	Real-World Data
Tofacitinib, Upadacitinib, Filgotinib (JAK inhibitors)	Black-box warning for MACE; use only if no other treatment is available in patients ≥ 65 years, smokers, or with CV risk factors.	OCTAVE: ACSs occurred infrequently; rare arrhythmic events mainly in higher doses [79]; ORAL Surveillance (RA): ↑MI risk with tofacitinib vs anti-TNF no ↑MACE overall; signal with high doses possible [81]	MACE and HF rate not different vs anti-TNF in IBD patients ≥ 50 years [78]
Ozanimod, Etrasimod (S1P modulators)	Contraindicated in recent MI/unstable angina, NYHA III/IV HF, severe untreated AV block without pacemaker; recommend baseline ECG for patients with bradycardia or conduction disorders.	TRUE NORTH (ozanimod): no HF events; 2 cases asymptomatic bradycardia; ELEVATE UC trials (etrasimod): rare, transient bradycardia or AV block; no Mobitz II or serious arrhythmias; no ACS events due to trial exclusion criteria [84,88]	Ozanimod: real-world 12-month data: no HF, ACSs, or symptomatic bradycardia; Etrasimod: limited real-world data [87]

CV, cardiovascular; HF, heart failure; NYHA: New York Heart Association; Mi, myocardial infarction; AV: atrioventricular; IBD: inflammatory bowel disease; ACSs, acute coronary syndromes; AF, atrial fibrillation; SPC, Summary of Product Characteristics.

## Abbreviations

The following abbreviations are used in this manuscript:

ACS	Acute Coronary Syndrome
AF	Atrial Fibrillation
AV	Atrioventricular
BP	Blood Pressure
CAD	Coronary Artery Disease
CD	Crohn’s Disease
CV	Cardiovascular
FAERS	FDA Adverse Event Reporting System
FDA	Food and Drug Administration
GLS	Global Longitudinal Strain
HF	Heart Failure
HR	Heart Rate
IBD	Inflammatory Bowel Disease
IL	Interleukin
JAK	Janus Kinase
LVEF	Left Ventricular Ejection Fraction
MACE	Major Adverse Cardiovascular Events
MI	Myocardial Infarction
NSTEMI	ST-elevation Myocardial Infarction
NT-proBNP	N-terminal pro-B-type Natriuretic Peptide
NYHA	New York Heart Association
RA	Rheumatoid Arthritis
RCT	Randomized Controlled Trial
S1P	Sphingosine-1-Phosphate
SPC	Summary of Product Characteristics
UC	Ulcerative Colitis
